# Vanadium in Bipolar Disorders—Reviving an Old Hypothesis

**DOI:** 10.3390/ijms232213901

**Published:** 2022-11-11

**Authors:** Vishnu Priya Sampath, Shiv Vardan Singh, Ilana Pelov, Noa Horesh, Hiba Zannadeh, Ofir Tirosh, Yigal Erel, David Lichtstein

**Affiliations:** 1 Department of Medical Neurobiology, Institute for Medical Research Israel-Canada, Faculty of Medicine, The Hebrew University of Jerusalem, Jerusalem 91905, Israel; 2 Jerusalem Mental Health Center, Eitanim Psychiatric Hospital, Jerusalem 91060, Israel; 3 The Fredy and Nadine Herrmann Institute of Earth Sciences, Faculty of Science, The Hebrew University of Jerusalem, Jerusalem 91904, Israel

**Keywords:** Bipolar disorder, vanadium, brain, serum, behavior, Na^+^, K^+^-ATPase

## Abstract

Bipolar disorder (BD) is a severe and common chronic mental illness. The biological basis of the disease is poorly understood and its treatment is unsatisfactory. Our previous studies supported the notion that alterations in Na^+^, K^+^-ATPase activity were involved in the etiology of BD. As various chemical elements inhibit Na^+^, K^+^-ATPase, we determined the concentration of 26 elements in the serum of BD patients before and after treatment and in postmortem brain samples from BD patients, and compared them with matched controls. The only element that was reduced significantly in the serum following treatment was vanadium (V). Furthermore, the concentration of V was significantly lower in the pre-frontal cortex of BD patients compared with that of the controls. Intracerebroventricular administration of V in mice elicited anxiolytic and depressive activities, concomitantly inhibited brain Na^+^, K^+^-ATPase activity, and increased extracellular signal-regulated kinase phosphorylation. A hypothesis associating V with BD was set forth decades ago but eventually faded out. Our results are in accord with the hypothesis and advocate for a thorough examination of the possible involvement of chemical elements, V in particular, in BD.

## 1. Introduction

Depressive disorders and Bipolar disorder (BD), are a serious and devastating group of diseases. Affecting some 10% of the population, they pose a significant public health issue. These disorders are manifested by a combination of symptoms that interfere with the ability to work, study, sleep, eat, and enjoy pleasurable activities. Depressive illnesses, and more so Bipolar and related disorders, have a strong genetic component. A large number of genes have been suggested to be involved in BD, and environmental factors, such as stress, are considered important as well [1,2].

The etiology of BD remains to be elucidated. In the past five decades, focus on the brain monoaminergic systems, which contain serotonin, norepinephrine, and dopamine (the monoaminergic hypothesis), has dominated the biological approach to the study and treatment of depression [3], leading to the development of drugs for its treatment [4]. However, the hypothesis does not take into account the time lag between the increase in monoamine levels, which occurs within minutes, and the alleviation of depressive symptoms, which require weeks of continued drug exposure [5]. Therefore, the monoaminergic hypothesis in itself cannot account for the psychopathology of depressive disorders [6], and studies on the mechanisms underlying these maladies focus on other neurotransmitter and neuropeptide systems, and their interplay. To this end, it was suggested that a dysregulated hypothalamic-pituitary-adrenal axis played a role in the pathophysiology of depressive disorders [7]. Cortisol, corticotrophin-releasing hormone, and adrenocorticotropic hormone, as well as amino acid neurotransmitters such as glutamic acid and gamma aminobutyric acid [8,9], brain-derived neurotrophic factor [10] and mitochondrial function [11] are considered key participants in the etiology of these diseases. 

Vanadium is a widely distributed element, and its biological actions have been studied extensively [12,13]. The average human diet provides 10–160 mg of V a day, mainly from mushrooms, seafood, black pepper, parsley, fennel seeds, grains, and spinach [13,14]. After entering the bloodstream, V compounds are converted into vanadyl cations, which form complexes with transferrin and ferritin and, less frequently, albumin, hemoglobin, and low molecular weight plasma components (citrate, lactate, and phosphate) [15]. In human serum, the V concentration of healthy individuals ranges from 1–100 nM [16]. V is distributed differentially in various organs, accumulating mainly in the kidney and, to a lesser extent, in the liver, bones, spleen and brain [14,15]. Vanadate (+5) and/or vanadyl (+4) ions are very potent inhibitors of Na^+^, K^+^-ATPase and other ATPases, phosphatases and kinases, and are activators of adenylate kinase, glucose-6-phosphate dehydrogenase, and insulin receptor [15,17,18]. The pharmacological effects of V compounds have been studied extensively, particularly in relation to their insulin-mimetic [13,18,19,20] and anti-cancer [18,21] effects. For detailed descriptions of vanadium chemistry and pharmacology see [13,17].

The pioneering demonstration by Cantley in 1977 [22] showed that V was a potent reversible inhibitor of Na^+^, K^+^-ATPase, and the established important role of this enzyme in neuronal function spurred interest regarding possible involvement of this ion in brain diseases. In the following 10 years, several articles, mainly by Naylor and his colleagues, suggested a link between V and psychotic disorders, particularly its involvement in the etiology of BD. Increased plasma concentrations of V were reported in mania and depression [23,24] and raised hair levels were found in mania [25]; V levels in the hair and serum of depressed patients decreased following recovery [26]; Both manic and depressed patients significantly improved with reduced V intake [27]; Therapies based on decreasing the V levels in the body (e.g., ascorbic acid, EDTA, methylene blue) have been reported to be effective in both depression and mania [25]; Lithium, the first-line medicine in the treatment of BD, has been reported to reduce the inhibition of Na^+^, K^+^-ATPase by vanadate [28], thus linking V and Na^+^, K^+^-ATPase inhibition to BD. Despite these remarkable observations, the hypothesis regarding the involvement of V ions in BD faded out and is, to the best of our knowledge, not addressed experimentally and scarcely mentioned in the relevant literature [29].

In the present study, we show that V concentrations were reduced in the serum of BD patients following treatment, and their concentrations were lower in postmortem PFC samples from BD patients compared with those of psychiatrically healthy individuals. In addition, we show that the intracerebroventricular (*i.c.v.*) injection of sodium metavanadate (NaVO_3_) into mice elicited depressive-like behavior in the forced swimming tests, concomitantly inhibited in vivo Na^+^, K^+^-ATPase activity, and increased ERK phosphorylation.

## 2. Results

### 2.1. Serum V Is Decreased Following Treatment of BD Patients

**Figure 1:**
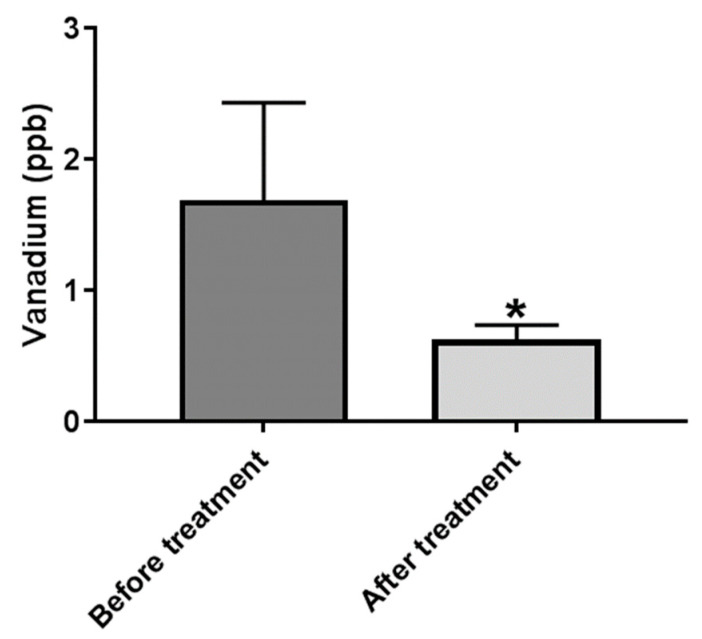
Vanadium concentration in the sera of BD patients before and after treatment. Blood samples were collected from the patients immediately upon admission due to a manic episode to Psychiatric Hospital. A second blood sample was collected 2–3 weeks later following initial stabilization and partial remission of the symptoms. Vanadium concentrations were determined in sera samples as described in Materials and Methods. The results are expressed as mean ± SEM, *n* = 11. The difference between the groups was analyzed using nonparametric Exact One-tail Wilcoxon Signed-Ranks Test. * significantly lower than before treatment, *p* 0.05.

Considering the hypothesis that V is involved in the etiology of BD, and that increased serum V concentration is associated with the disease, we first addressed the possibility that V concentrations may be reduced following treatment. Blood samples were collected from 11 patients immediately upon their admission to the acute psychiatric units of the Eitanim Psychiatric Hospital (Eitanim, Israel), due to a manic psychotic episode. All the patients were females, average age 35.36 ± 12.96 (S.D) years, and were treated initially with clothiapine, or clonazepam, or lorazepam, or a combination of these drugs, with mood stabilizers like lithium and/or antipsychotic drugs, which were added after the first sampling of the blood. A second blood sample was collected 2–3 weeks later, after the patient had stabilized and been released from the acute inpatient unit or the hospital. Mood and behavior stabilization, manifested by longer sleep periods, reduced grandiose delusions, and reduced irritability and talkativeness, was determined by the professional medical staff. The samples were extracted and element concentrations were determined as described in the Materials and Methods. Vanadium concentrations in the sera of the patients, before and after treatment, are depicted in Figure 1.

The concentration of all other tested elements in the sera are not presented here and will be published separately [30]. Since the distributions of the concentration values were not normal, and the sample size was small (11 pairs), a nonparametric test, the Exact One-Tailed Wilcoxon Matched-Pairs Signed Ranks Test, was used to evaluate the effect of the treatment. Among the elements measured, only the concentration of V changed significantly following treatment (*p* = 0.034).

### 2.2. Vanadium Concentrations in PFC of BD Patients Is Lower than in Matched Controls

The reduction in V in the serum of manic BD patients following treatment prompted us to determine V in postmortem brain samples of BD patients and matched controls. To this end, freshly frozen PFC tissues were obtained from the human postmortem brain tissue collection at the Human Brain Collection Core, at the National Institute of Mental Health (NIMH). The demographic characteristics of the control and BD patients’ brain samples—gender, cause of death, use of alcohol, mood stabilizers, and psychoactive drugs—are presented elsewhere [30]. According to the analysis of variance (ANOVA), the groups did not differ in postmortem interval, brain weight, or pH. Chi square analysis indicated that the groups did not differ in terms of gender. Most of the studied BD patients used psychoactive drugs, and all of them committed suicide, whereas only 25% of the control group used psychoactive drugs, and none of them committed suicide.

**Figure 2:**
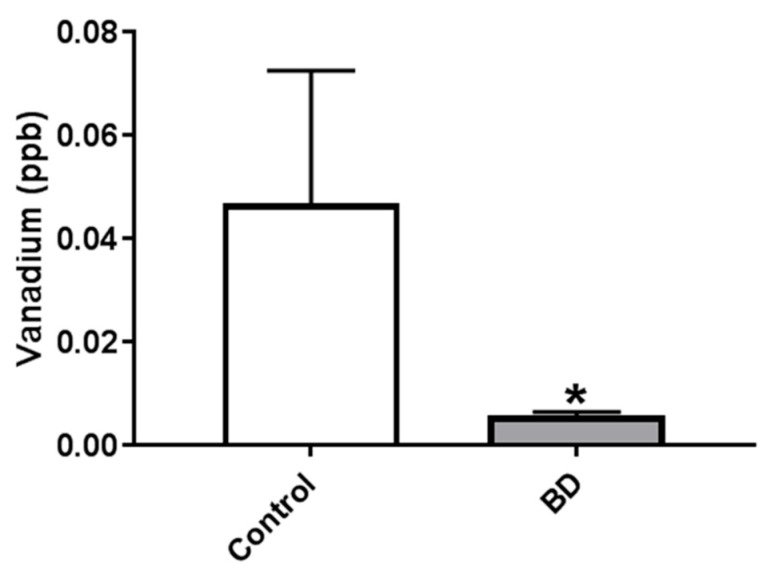
Vanadium concentrations in PFC of BD patients and matched controls. PFC brain samples were obtained from the human postmortem brain tissue collection at the Human Brain Collection Core, National Institute of Mental Health (NIMH). Vanadium concentrations were determined as described in Materials and Methods. Results are expressed as mean ± SEM, *n* = 20. The difference between the groups was analyzed utilizing nonparametric Exact One-tail Mann–Whitney Test. * significantly lower than control samples, *p* 0.05.

A total of 5–10 mg of wet brain tissue samples were extracted and element concentrations were determined, as described in the Materials and Methods. Vanadium concentrations in BD patients were significantly lower in the PFC of BD patients compared to the matched controls (Figure 2). The concentrations of additional five elements were found to be lower in the BD patients [30].

### 2.3. Vanadium Induces Depression-like Behavior in Mice

The observations described above, on the difference in V brain concentrations between BD and control subjects and their reduction in the serum of BD patients following treatment, spurred us to examine some of the V biological effects in vivo. Although many biological consequences have been attributed to V (see above), the effects of the direct administration of V into the brain on behavior, has, to the best of our knowledge, not been investigated.

**Figure 3:**
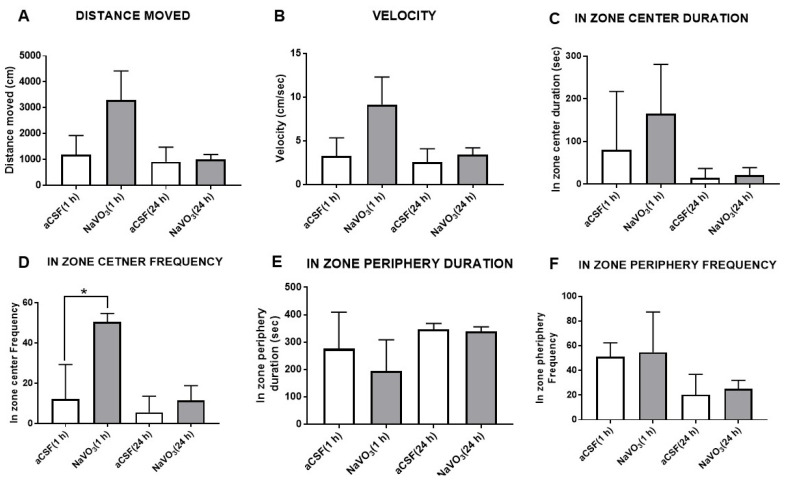
Effect of NaVO_3_ on mice behavior in open field test. NaVO_3_ was injected (3 µL from a 10 mM stock solution in aCSF) into the lateral ventricle. OFT was performed as described in Materials and Methods. Distance moved (**A**), velocity (**B**), in-zone duration (**C**), in-zone center frequency (**D**), in-zone periphery duration (**E**), and in-zone periphery frequency (**F**) were recorded with EthoVision XT, video tracking software. (number of mice = 6); * significantly different from mice receiving aCSF, *p* 0.05.

The effect of NaVO_3_ administration (*i.c.v*., 3 μL from a 10 mM solution) in the aCSF of mice on their behavior in the open field test (OFT) is depicted in Figure 3. The treatment did not significantly affect parameters of mouse behavior, including the distance and velocity of movement (Figure 3A,B) and the time spent in the field center or periphery (Figure 3C,E,F). The lack of change in activity in the OFT was observed 1 h and 24 h after NaVO_3_ injection. A significant increase in in-zone center frequency was observed only one hour after V administration (Figure 3D), which was indicative of anxiolytic activity.

**Figure 4:**
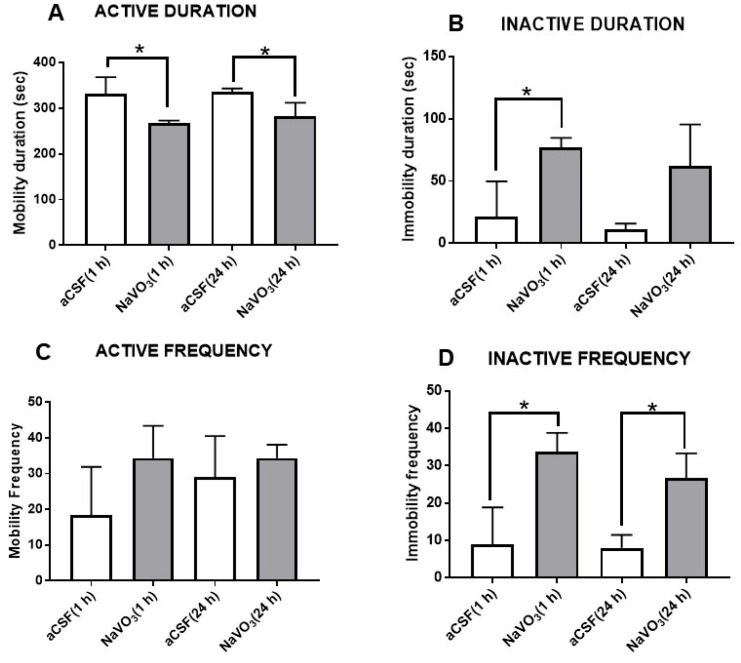
Effect of NaVO_3_ on mouse behavior in forced swimming test. NaVO_3_ was injected (3 µL from a 10 mM stock solution in aCSF) into the lateral ventricle. The FST was performed as described in Materials and Methods. Active duration (**A**), inactive duration (**B**), active frequency (**C**), and inactive frequency (**D**) were recorded with EthoVision XT, video tracking software. (number of mice = 6); * significantly different from mice receiving aCSF, *p* 0.05.

The effect of NaVO3 on behavior was also tested in the forced swimming test (FST), an animal model of depression [31]. Interestingly, the administration of the compound caused significant “depression-like” behavior, manifested by a decrease in activity duration (Figure 4A) and an increase in duration of inactivity (Figure 4B) and inactive frequency (Figure 3D). The effects on active duration time and inactive frequency were observed 1 h as well as 24 h after NaVO_3_ administration (Figure 4).

### 2.4. Vanadium Inhibits Na^+^, K^+^-ATPase Activity In Vivo

**Figure 5:**
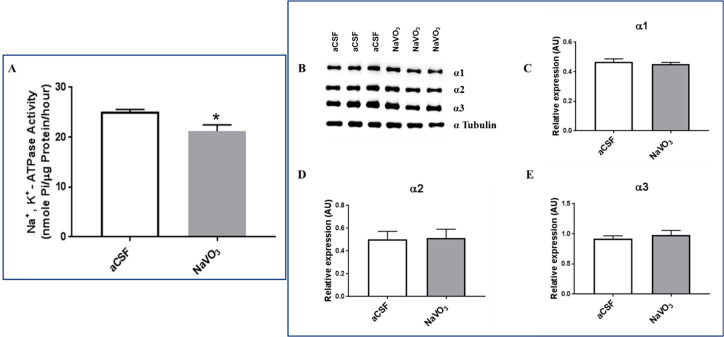
Effect of NaVO_3_ administration on brain Na^+^, K^+^-ATPase activity and Na^+^, K^+^ ATPase α isoform expression. Mice were treated with NaVO_3_ (3 µL from a 10 mM stock solution in aCSF) and their behavior was examined as described in the legend to Figure 3 and Figure 4. Following the behavioral tests (Figure 4) the mice were euthanized. The brains were exposed and the frontal cortex was dissected. A microsomal fraction was prepared and Na^+^, K^+^-ATPase activity was determined as described in Materials and Methods. Na^+^, K^+^-ATPase activity (**A**) is expressed as the mean ± SEM (*n* = 6). Na^+^, K^+^-ATPase activity in the control mice (25.72 nmol Pi/μg protein/h) represented 35% of the total ATPase activity (71.94 nmol Pi/μg protein/h) in this membrane preparation. Na^+^, K^+^-ATPase α subunit isoform expression was determined by western blot analysis as described in Materials and Methods. (**A**): Representative western blots. (**B**): Quantitative α isoform expression (**C**–**E**) values were normalized to the levels of α Tubulin (*n* = 4). * significantly lower than in mice receiving aCSF, *p* 0.005.

In view of the behavioral effects induced by the *i.c.v.* administration of V, we examined several biochemical parameters known to be affected by V under these experimental conditions. As V is a well-established inhibitor of Na^+^, K^+^-ATPase [22], we first examined the effect of the V treatment on this parameter. As is shown in Figure 5A, as expected, Na^+^, K^+^-ATPase activity was significantly reduced (16%) in the brains of mice that received NaVO_3_ compared with those of mice receiving aCSF.

The reduction in Na^+^, K^+^-ATPase activity can result from reduced ATP hydrolysis and ion transport or a decrease in the amount of the protein in the tissue. To address this issue, we determined the three α isoforms of Na^+^, K^+^-ATPase activity in the brain following V treatment. As shown in Figure 5B–E, V did not affect the levels of any of the three α isoforms under our experimental condition. It can therefore be concluded that the reduction in Na^+^, K^+^-ATPase activity was due to the direct inhibition of transport by the element.

### 2.5. Vanadium Increases ERK but Not AKT Phosphorylation In Vivo

The effects of V on behavior may be mediated by changes in intracellular signaling. It is well established that Na^+^, K^+^-ATPase inhibitors, such as ouabain or digoxin, activate extracellular signal-regulated kinase (ERK) and protein kinase B (AKT).

**Figure 6:**
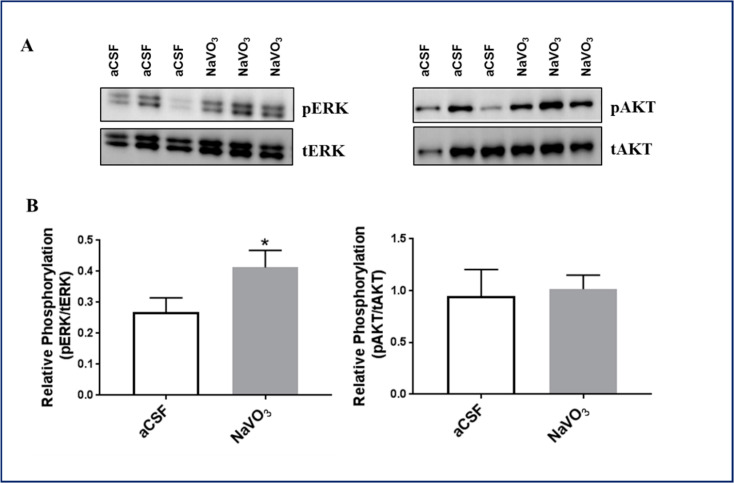
Effect of NaVO_3_ administration on brain ERK and AKT phosphorylation. Mice were treated with NaVO_3_ (3 µL from a 10 mM stock in CSF) and their behavior was examined as described in the legend to Figure 3 and Figure 4. Following the behavioral tests (Figure 4) the mice were euthanized. The brains were dissected and the proteins were extracted. ERK and AKT phosphorylation was determined by western blot analysis, as described in Materials and Methods. (**A**): Representative western blots. (**B**): Quantitative ERK and AKT relative phosphorylation. The values were normalized to the levels of Total ERK and AKT levels. The values are expressed as the mean ± SEM (*n* = 4). * significantly different from mice treated with aCSF, *p* 0.05.

We therefore tested whether V administration also affected ERK and AKT phosphorylation. As shown in Figure 6A,B, the *i.c.v.* administration of V elicited a significant increase in ERK (65%), but not in AKT phosphorylation under our experimental conditions.

## 3. Discussion

For the past decade, we and others presented evidence for the involvement of endogenous Na^+^, K^+^-ATPase inhibitors in BD [32]. It is well recognized that numerous elements bind to and affect Na^+^, K^+^-ATPase activity. These include Na^+^, K^+^, and Mg^++^, the substrates for Na^+^, K^+^-ATPase function [33], as well as other elements, including Al, Ca^++^, Co, Cu, Pb, Hg, V, and Zn [22,34,35,36,37]. We raised the hypothesis that the inhibition of Na^+^, K^+^-ATPase by any of these or other elements may be a contributing factor in the etiology of BD. The hypothesis, outlined in the introduction, relating to the involvement of V, a well-established Na^+^, K^+^-ATPase inhibitor, in the etiology of BD, is in accord with this notion. To address this issue, we first measured the concentration of V and other elements by using state of the art methodology; ICP-MS measurements followed clean-lab protocols, in the serum of BD patients, before and after treatment. Astonishingly, of the 20 elements tested, only the V concentrations were significantly reduced in the sera after treatment (Figure 1). This was in complete agreement with the results of Naylor and colleagues, who demonstrated, 40 years ago by using neutron activation analysis, that the V concentration in the serum of depressed patients decreased following recovery [26]. As V is an element, there is no synthesis or degradation in the body, and the reduction in the serum was a consequence of either decreased dietary intake or increased excretion in the urine or feces. An additional possibility is that V was concentrated in the tissue of BD patients, thereby decreasing the circulating levels. To address these possibilities, a thorough study on V pharmacokinetics in control and BD patients should be performed.

The observation that serum V concentration was reduced following the treatment of BD patients prompted us to analyze V and other element concentrations in the postmortem brain samples of BD patients compared with that of psychiatrically healthy controls. A detailed analysis of the element concentrations and element inter-correlations will be published separately [30].

We found that the concentrations of V were significantly lower in the brains of BD patients compared with those of the control (Figure 2). Importantly, since all BD patients were treated with various medications, the lower V levels in their brains were in accord with the lower levels in the serum following treatment. The reason for these lower concentrations of V in the brains of BD patients is not known. It is noteworthy that the element balance within the brain is regulated in a complex manner by brain barrier systems [38] and their homeostasis relies on the processes of absorption, distribution, biotransformation, and excretion [39]. Hence, alteration of one of these processes, due to genetic or environmental causes, may be the underlying mechanism for the reduced V concentrations in BD.

The lower V concentrations in the brains of BD patients may be closely involved in the etiology of the disease, or result from the illness. Our study cannot discriminate between these possibilities. However, if V is involved in the cause of the disease, one would expect that the administration of V into the brain would induce biochemical and behavioral changes. We therefore tested the effect of *i.c.v.* administration of NaVO_3_ into mice. As expected, we found that the administration of V affected behavior (Figure 3 and Figure 4), concomitantly inhibited Na^+^, K^+^-ATPase activity (Figure 5), and activated ERK signaling (Figure 6A). Although the effect of the administration of V into the brain on behavior was not explored previously, our results were in consonance with reports that tested the effect of an increase in V in the circulation, following increased dietary intake, on behavior. They claimed that the acute oral administration of NaVO_3_ induced cognitive decline and behavioral impairments in mice [40] and chronic (8 weeks) V treatment in rats resulted in a reduction in motor activity in the OFT and in impaired learning ability [41]. The inhibition of Na^+^, K^+^-ATPase activity and changes in ERK phosphorylation observed following NaVO_3_ administration were in agreement with numerous previous studies demonstrating the wide range of effects of V (see Introduction). The following sequence of events may be suggested: a decrease in V in the brain (observed in BD patients, Figure 2) caused an increase in Na^+^, K^+^-ATPase activity (Figure 5) and reduction in ERK activity (Figure 6). These changes may have caused cellular alterations (i.e., hyperpolarization, gene expression) which may lead deviations in behavior. It is tempting to speculate that these biochemical changes are responsible for the observed behavioral effects of the compound and its possible involvement in BD. Cumulatively, our results support the hypothesis that an imbalance in V is a factor in the etiology of BD, and we advocate for an in-depth investigation of this hypothesis.

## 4. Materials and Methods

### 4.1. Serum from BD Patients

The study on human serum samples was approved by the Helsinki Committees of Eitanim and Hadassah Hospitals, in Jerusalem. We recruited patients over 18 years of age, who had developed severe manic symptoms and were admitted to the Emergency Room at the Eitanim Psychiatric Hospital (Eitanim, Israel). A total of 11 female patients were recruited between the years 2018 and 2019. The diagnosis of a manic episode was performed by the treating psychiatrists and verified by a staff psychiatrist from the research team. Blood samples (10 mL) were collected from the patients immediately upon admission. A second blood sample (10 mL) was collected 2–3 weeks later following initial stabilization and partial remission of the symptoms, which enabled the patients to give written informed consent for participation in the study. First, samples from patients that did not give their consent to participate in the study were discarded. The samples were centrifuged (4 °C, 2000× *g*, 15 min) to obtain sera, which were frozen and stored at −80 °C until use.

### 4.2. Brain Postmortem Samples

All postmortem human brain tissue samples used in this study were obtained from the Human Brain Collection Core (HBCC), Intramural Research Program, of the NIMH, NIH (http://www.nimh.nih.gov/hbcc, accessed on 5 January 2019), in Bethesda, MD, USA. Samples from 20 BD patients and 20 controls were analyzed. The demographic and clinical characteristics of this cohort are shown in Table 3 in the adjacent paper [30]. The two groups were matched for several clinical variables. According to analysis of variance (ANOVA), the groups did not differ in age or postmortem interval, brain weight, or pH. Chi square analysis indicated that the groups did not differ in terms of gender. The NIMH received ethics approval for the brain collection.

### 4.3. Determination of Elements in Serum and Brain Tissue

Sample preparation for elemental analysis was carried out in a clean laboratory in the Institute of Earth Sciences at the Hebrew University. The clean-laboratory includes a monitored positive pressure air supply with HEPA filtration and entirely non-metallic construction. A total of 5–10 mg of wet brain tissue and 5 mL of sera was extracted in pre-conditioned PFA, and dissolved in Teflon beakers containing high purity nitric acid (HNO_3_) and hydrogen peroxide (H_2_O_2_) (2 mL 70% HNO_3_ + 1 mL 30% H_2_O_2_). After complete dissolution, the samples were dried almost completely, re-dissolved in 1% high purity HNO_3_, and diluted in triple distilled water (18 Ω) to the desired volume for analysis. The concentration of 26 elements (Li, Na, K, Rb, Be, Mg, Ca, Sr, Ba, V, Cr, Mn, Fe, Co, Ni, Cu, Zn, Mo, Cd, B, Al, Pb, Bi, U, P (as PO_4_), S (as SO_4_)) was determined with Inductively Coupled Plasma Mass Spectrometry (ICP-MS) (Agilent 7500 cx and Agilent 8900). The ICP-MS was calibrated with a series of multi-element standard solutions (Merck ME VI) and a blank (triple distilled water). All elements were measured using a collision cell (He at 5 mL/min). A solution of internal standards (50 μg/L Sc, 5 μg/L Re, and 5 μg/L Rh) was injected alongside the samples during the analytical session for drift correction. The USGS SRMs (T-235, T-221; in dilute HNO_3_ matrix) were examined after calibration for accuracy assessment. Precision, determined by multiple runs of 1–2 standards, was estimated as 5%. The results were expressed as the mean ± SE of 20 brain samples, or 11 serum samples.

### 4.4. Animals and Behavioral Test

All procedures were carried out in accordance with the Israel Ministry of Health Regulations and were approved by The Hebrew University of Jerusalem Animal Care and Use Committee (Protocol #MD-1815–4). Male BALB/c mice (Harlan, Jerusalem, Israel), aged 7–8 weeks, were housed in the Hebrew University Animal Facility in a temperature-controlled SPF facility (22 ± 2 °C), with a 12 h/12 h light–dark cycle, and with food and water provided *ad libitum*. The surgical procedure and behavioral tests were performed as previously described [42]. Briefly, mice were divided randomly into two groups—control and vanadate-treated. The mice received an *i.c.v.* injection of 3 µL of artificial cerebrospinal fluid (aCSF) or sodium metavanadate (NaVO_3_) in aCSF (10 mM) into the lateral ventricle. After 15 min, each mouse was placed in a 50 × 50 cm open-field arena, which it was allowed to explore for 6 min. The behavioral tests were performed during the light phase (10:00–14:00) under dimmed halogen lights (~35 Lux), as described previously [43]. Activities were monitored and quantified with an automated camera-based computer tracking system Ethovision XT (Noldus Information Technologies, Wageningen, The Netherlands). The mice were placed in a water tank and their swimming was monitored for 6 min, as previously described [44]. The behavioral tests were repeated after 24 h. The mice were then sacrificed, and the brains were dissected and frozen (−80 °C) for later analysis.

### 4.5. ATPase Activity

The mouse brain microsomal fraction was prepared as previously described [45]. Na^+^, K^+^-ATPase activity in the microsomal fraction was determined by the amount of inorganic phosphate released during incubation at 37 °C, as previously described [45]. In brief: A 100 μL volume of a microsomal preparation (50–100 pg protein) was added to an 800 μL reaction buffer (50 mM Tris-Base, 120 mM NaCl, 10 mM KCI, 4 mM MgCl_2_, pH 7.4) in the presence of varying concentrations of an inhibitor (bufalin or bufalin derivatives). Following 1 hr of incubation, 10 μL of ATP (2 mM final concentration) were added, and the incubation was continued for an additional hr. The reaction was terminated by the addition of 200 μL of 30% trichloroacetic acid, and the tubes were placed on ice for 10 min. Following centrifugation (500× *g*, 10 min, 4 °C), 750 μL of the supernatant were removed for the colorimetric determination of inorganic phosphate, as described previously [46].

### 4.6. Quantification of Na^+^, K^+^-ATPase α Isoform, ERK and AKT Phosphorylated Protein by Western Blot Analysis

The brain tissues were thawed. The PFC was dissected and homogenized in radio-immunoprecipitation (RIPA) buffer and Protease Inhibitor Cocktail (Sigma-Aldrich, St. Louis, MO, USA) and centrifuged (14,000× *g*). The protein content of the supernatants was quantified with the Bio-Rad Protein Assay (Bio-Rad Laboratories, Hercules, CA, USA), according to the manufacturer’s instructions. The samples were subjected to western blot analysis, as previously described [43]. The following primary antibodies were used: mouse monoclonal anti-Na^+^, K^+^-ATPase-α1 subunit antibody (1:800) (Merck, Kenilworth, NJ, USA); rabbit polyclonal anti-Na^+^, K^+^-ATPase-α2 subunit antibody (1:3000), which was received from Prof. Thomas Pressley (Texas Tech University, Lubbock, TX, USA); mouse monoclonal anti-Na^+^, K^+^-ATPase-α3 subunit antibody (1:4000); and mouse monoclonal α Tubulin antibody (1:800) (Invitrogen and Sigma-Aldrich), as well as phospho-p44/42 (ERK1/2 Tyr204) rabbit monoclonal antibody (1:800), phospho-Akt (Ser473) and rabbit monoclonal antibody (1:800) (Cell Signaling Technology, MA, USA). The membranes were incubated with horseradish peroxidase-conjugated secondary antibodies (Jackson Immuno Research Laboratories, West Grove, PA, USA). The band intensity was detected with a chemiluminescence system (Fusionsolo S, VILBER, Collégien, France), and quantified according to image J.

### 4.7. Statistical Analyses

The demographic and clinical characteristics of the post-mortem brain samples from control and BD subjects were analyzed with two-way analysis of variance (ANOVA). Chi-square analysis was used to detect gender differences. The nonparametric Exact One-tailed Wilcoxon Matched-Pairs Signed Ranks Test was used to evaluate the differences between pairs. For V concentrations in the brain, the results were expressed as the median, and the exact one-tailed Mann–Whitney test was used. Mouse behavior and changes in western blots for ERK and AKT were analyzed with the unpaired two-tailed t-test, and brain Na^+^, K^+^-ATPase activity was analyzed with a one-tailed unpaired t-test. All the values were expressed as the mean ± SE, and all analyses were performed with GraphPad Prism v 7.03 (GraphPad Software, Inc., San Diego, CA, USA), where *p* 0.05 was considered significant.

## Data Availability

Not applicable.

## Abbreviations

BD, Bipolar disease; PFC, Prefrontal cortex; HBCC, Human Brain Collection Core; ICP-MS, Inductively coupled plasma mass spectrometry; rs, Spearman’s correlation coefficient; ICV, intracerebroventricular; OFT, Open field test; FST, Forced swimming test; Na^+,^ K^+^-ATPase, Sodium, Potassium-activated Adenosine Triphosphatase; ERK, Extracellular signal-regulated kinase; AKT, protein kinase B.
